# NMR Metabolite Profiles of the Bivalve Mollusc *Mytilus galloprovincialis* Before and After Immune Stimulation With *Vibrio splendidus*

**DOI:** 10.3389/fmolb.2021.686770

**Published:** 2021-09-03

**Authors:** Riccardo Frizzo, Enrico Bortoletto, Tobia Riello, Luigi Leanza, Elisabetta Schievano, Paola Venier, Stefano Mammi

**Affiliations:** ^1^ Department of Chemical Sciences, University of Padova, Padova, Italy; ^2^ Department of Biology, University of Padova, Padova, Italy

**Keywords:** *Mytilus galloprovincialis*, *Vibrio splendidus*, NMR, metabolomics, immune stimulation, anaerobiosis, flow cytometry, immunity

## Abstract

The hemolymph metabolome of *Mytilus galloprovincialis* injected with live *Vibrio splendidus* bacteria was analyzed by ^1^H-NMR spectrometry. Changes in spectral hemolymph profiles were already detected after mussel acclimation (3 days at 18 or 25 °C). A significant decrease of succinic acid was accompanied by an increase of most free amino acids, mytilitol, and, to a smaller degree, osmolytes. These metabolic changes are consistent with effective osmoregulation, and the restart of aerobic respiration after the functional anaerobiosis occurred during transport. The injection of *Vibrio splendidus* in mussels acclimated at 18°C caused a significant decrease of several amino acids, sugars, and unassigned chemical species, more pronounced at 24 than at 12 h postinjection. Correlation heatmaps indicated dynamic metabolic adjustments and the relevance of protein turnover in maintaining the homeostasis during the response to stressful stimuli. This study confirms NMR-based metabolomics as a feasible analytical approach complementary to other omics techniques in the investigation of the functional mussel responses to environmental challenges.

## Introduction

Bivalvia comprise many thousands of marine and freshwater species, among which mussels, oysters, and clams are the most relevant in aquaculture (Wijsman et al., 2019). These bivalves also represent interesting models for studying innate immune responses and host–pathogen interactions since they are commonly exposed to microscopic pathogens such as viruses, bacteria, and parasites (Fernández Robledo et al., 2019). The innate immune system of bivalves consists of cellular and humoral components that operate in concert against invading microorganisms. Once activated by pathogen-associated molecular patterns (PAMPs), the hemocytes circulating in a system of vessels and open sinuses can perform chemotaxis, phagocytosis and oxidative burst, and encapsulation, and quickly release antimicrobial peptides (AMPs), enzymes, and proteins essential as an early defense front line (Gerdol et al., 2018). Also, the PAMP-induced release of AMPs from granular hemocytes supports pathogen killing in the so-called extracellular networks (ETs) (Poirier et al., 2014; Romero et al., 2020). While adding further layers of complexity, the evolutionary expansion of immune-related genes and the unexpectedly significant phenomenon of gene presence–absence variation in *Mytilus galloprovincialis* greatly enhances and modulates the host resistance to pathogens (Takeuchi et al., 2016; Gerdol et al., 2020).

Gram-negative and facultatively anaerobic *Vibrio* bacteria are widespread in the aquatic environments, and species such as *Vibrio anguillarum*, *Vibrio coralliilyticus*, *Vibrio splendidus*, *Vibrio aestuarianus*, and *Vibrio mediterranei* are known to induce severe vibriosis that can occasionally escalate to episodes of bivalve mass mortality (Vezzulli et al., 2010; Sun et al., 2014; Green et al., 2019; Andree et al., 2021; Garcia et al., 2021). Specifically, the strain *V. splendidus* LGP32 is regarded as a pathogen owing to its association to oysters during summer mortality events and the demonstration of a remarkable virulence in laboratory trials with oysters and clams (Roux et al., 2009).

Intriguingly, *Mytilus spp*., seem to be resistant to *Vibrio*-induced mortality (Romero et al., 2014). Experimental treatments of *Mytilus spp.* with *Vibrio splendidus* LGP32 have been reported in several *in vivo* and *in vitro* studies, which investigated the dynamics of cellular responses (Balbi et al., 2013; Parisi et al., 2019) and gene expression changes in hemocytes and tissues (Tanguy et al., 2018; Rey-Campos et al., 2019b; Saco et al., 2020), also exploring the effects of repeated *in vivo* contacts with *V. splendidus* (Rey-Campos et al., 2019a)*.*

The exposure mode and strength of the stimulus certainly influence the mussel response to *Vibrio splendidus* LGP32. The injection of 10^7^ heat-killed *V. splendidus* cells through a hole created by shell filing significantly changed the constitutive transcript levels of AMPs, increasing defensin from three hpi to 3 days postinjection and causing a rapid decrease of myticin and mytilin with a minimum at 12 hpi in SW at 20°C (Cellura et al., 2007). Following the same injection mode, live *V. splendidus* caused a marked downregulation of AMPs at three hpi, partially reversed at 24 hpi, in the mussel hemolymph, while concurrent transcriptional changes revealed the upregulation of sensors (Toll-like and other receptors); NFkB and MAPK signaling elements; pro- and anti-apoptosis factors (AIF and IAP); inflammosome and immunoproteasome components; various hydrolases, proteases, and protease inhibitors; multifunctional calcium-binding proteins (calreticulin); membrane attack complex/perforin (MACPF) effector–mediating bacterial killing; protein chaperones (HSP70s); and glucose-regulated proteins (Venier et al., 2011). More recently, transcriptome sequencing highlighted myticins as the most expressed among other AMPs in the hemolymph of mussels injected with *V. splendidus* (10^7^ CFU, SW at 15°C) and, notably, a great interindividual variability of the analyzed transcriptomes, both in control and treated mussels. In particular, transcripts related to the metabolism of nucleotides, carbohydrates, cofactors, and vitamins were represented more in control mussels, whereas transcripts involved in innate immune responses (including neuropeptides and the immune-responsive gene one involved in the production of itaconic acid in the Krebs cycle), translation, and signal transduction (phosphatidylinositol, mTOR, and PI3K-Akt signaling pathways) were represented more in the infected mussels (Rey-Campos et al., 2019b; Sendra et al., 2020). Although few studies started investigating mussel miRNAs, still little is known about the posttranscriptional regulation of protein-coding transcripts in bivalves (Moreira et al., 2020; Rosani et al., 2021).

Overall, the study of the metabolic profiles of farmed bivalves is a promising, currently expanding, investigation field which promotes a better understanding of biological processes (Li et al., 2020). NMR spectroscopy offers a wide array of solutions to common metabolomic challenges; excellent reproducibility and nondestructive acquisition of spectra are fundamental features of this technique (Nagana Gowda and Raftery, 2019; Crook and Powers, 2020), which enables the monitoring of a biological matrix over time, before/after treatment, or simply its retrieval for conservation or further processing. Currently, this investigation approach helps understanding how aquatic organisms adjust their metabolism in response to environmental variation (Cappello, 2020). In bivalves, NMR-based metabolomics has been successfully applied to the study of host–pathogen interactions, with insights into the immune responses related to sex (Nguyen et al., 2018), different *Vibrio* strains (Liu et al., 2013), and pollution (François et al., 2015). Actually, a regular assessment of shellfish health and welfare is important to optimize farming practices and to certify product quality, and, therefore, a fast and well-calibrated NMR approach is an attractive practical strategy in fish and shellfish aquaculture (Roques et al., 2020).

In this study, we produced metabolic profiles of hemolymph and tissue extracts obtained from the Mediterranean mussel, *Mytilus galloprovincialis*, before and during a short-term immunostimulation trial with live *Vibrio* bacteria, thus gathering information from approximately 300 NMR spectra. As a result, we identified amino acids, typical osmolytes, organic acids and alcohols, and polyamines. These profiles are discussed in the light of the current knowledge on mussel and bivalve physiology.

## Materials and Methods

### Mussels and Tissue Sampling

More than 100 mussels (*Mytilus galloprovincialis* shell length 5.3 ± 0.5 cm) were collected near the Chioggia lagoon outlet, Italy (45°13’48.35”N 12°17’18.04”E; sampling date September 09, 2020; seawater (SW) temperature 24.6°C, pH 8.22, salinity 34‰, and dissolved oxygen 6.0 mg/L). The sampled bivalves were transported in a mildly refrigerated container (∼20°C) to the lab bench (∼26°C) in 3 h time and rapidly cleaned, before acclimatizing them in artificial SW at 35‰, pH 8.00 with continuous aeration, and daily feeding (Coral Food Xpro, Aquatic Nature, Belgium) for 2 days, as described in (Bortoletto et al., 2021). Each experimental group of mussels was separately maintained in 20 L tanks (max 30 mussels per tank) with daily water renewal. Mussels previously sampled (Sep 2019) were devoted to the methodological setup, whereas those sampled in 2020 were immunostimulated and analyzed as follows. Hemolymph was individually withdrawn, once only, with a syringe equipped with a 23G disposable needle from the posterior adductor muscle of *Vibrio*-injected mussels and paired controls (see below). Hemolymph was used in its entirety, as plasma fraction, or as hemocyte lysate. In detail, it was centrifuged at 800 × g for 15 min at 4°C, and the resulting supernatant (plasma) was carefully placed in a new Eppendorf tube on ice, whereas the pelleted hemocytes were resuspended and forced through a 26-G needle several times to obtain a hemocyte lysate. Other mussel tissues were individually dissected on ice to represent flesh (whole soft tissues), gills, and the digestive gland deprived of crystalline style. All biological matrices were snap-frozen in liquid nitrogen to block metabolic processes and enzymatic degradation before storage at −80°C.

### *Vibrio splendidus* Culture

The bacterial strain *V. splendidus* LGP32 was inoculated in Zobell Marine Broth 2216 (HiMedia Laboratories, Mumbai, India) and allowed to grow for 12–16 h at 25°C. Before use, the culture was refreshed by diluting it to 1:500 and further grown for 1–3 h. The concentration of bacteria was estimated by turbidimetry at 600 nm (Biospectrometer Basic, Eppendorf, Hamburg, Germany) based on a standard curve relating absorbance values to colony-forming units (CFUs).

### Mussel Immune Stimulation With Live *Vibrio* Cells and Hemolymph Sampling

**FIGURE 1:**
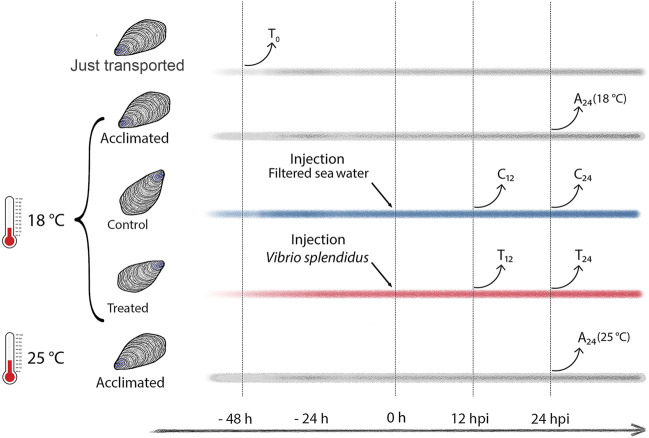
Study design (experimental mussel groups and hemolymph sampling time). Time lines in gray depict T_0_ (transported) mussels and the A_24_ (18°C) and A_24_ (25°C) mussels acclimated at 18°C and 25°C, respectively. Time lines in blue and red depict C_12_ and C_24_ mock-injected mussels, and T_12_ and T_24_ *Vibrio*-injected mussels, respectively, both sampled at 12 and 24 h postinjection (hpi).

Ten mussels per experimental condition were considered (details in Supplementary file 1). To test possible metabolic changes due to the mussel acclimation, a group of mussels was sampled immediately after transport to the laboratory (transport group, T_0_), and other two groups of mussels were sampled after 72 h of acclimation in artificial SW at 18°C (temperature routinely used in experimental infections) or at 25°C, namely, the SW temperature recorded at September 09, 2020 (acclimated groups A_24_ (18°C) and A_24_ (25°C), see Figure 1). The immune stimulation trial was performed as follows. Mussels were injected into the posterior adductor muscle either with 200 μL of filtered SW (mock controls, C_12_ and C_24_) or with 200 µL of 1 × 10^8^ CFU/mL of exponentially growing *V. splendidus* LGP32 (treated groups, T_12_ and T_24_). Hemolymph (at least 600 µL per mussel) was individually sampled, once only, at 12 or 24 h postinjection (hpi): a small aliquot was used for immediate hemocyte counting, whereas the remaining volume was snap-frozen in liquid nitrogen and stored at −80°C for NMR analysis. The bacterial load was evaluated on five additional mussels per experimental group by plating serial hemolymph dilutions (from 10^–2^ to 10^–6^) (Supplementary file 2) on Marine agar 2216 and thiosulfate-citrate-bile salt-sucrose (TCBS) Kobayashi agar (Biolife Italiana, Milano, Italy). We used the same five hemolymph samples to prepare fixed hemocytes (0.05% paraformaldehyde in filtered SW, 12–16 h) for a flow cytometry analysis and sorting.

### Flow Cytometry and Cell Sorting of Mussel Hemocytes

Formaldehyde-fixed hemocytes were analyzed by flow cytometry (BD FACSCANTO, Becton Dickinson, New Jersey, United States) with the parameters of the *forward scatter channel* (FSC) and the *side scatter channel* (SSC) set to 320 in a linear scale and to 230 in a logarithmic scale, respectively. For each sample, 10.000 and 20.000 events were recorded. The hemocyte subpopulations of hyalinocytes and granulocytes were sorted by an S3e cell sorter (Biorad, Hercules California United States), stained with 6% Giemsa (all chemical reagents from Sigma Aldrich, Steinheim, Germany, unless otherwise specified), and classified in bright field microscopy at 100x magnification.

### Tissue Processing and Acquisition of ^1^H 1D-NMR Spectra

A 500 µL aliquot of each (thawed) hemolymph or plasma fraction was prepared for the NMR analysis by adding 150 µL of a 0.8-mM trimethylsilylpropanoic acid (TSP) solution in D_2_O. The mussel tissues mentioned above were extracted according to the Folch method (Folch et al., 1957) using a 2:1 chloroform–methanol (v/v) solvent mixture at a solvent–tissue ratio of 8 mL/g. After addition of 4 mL deionized H_2_O and centrifugation at 5,400 × g for 15 min at 4°C, the methanol/water mixture was separated from the chloroform fraction. Both phases were evaporated and immediately stored at −20°C. All polar extracts were resuspended in 650 µL of D_2_O containing 0.8 mM TSP, transferred into a 5-mm NMR tube, and immediately analyzed. NMR spectra were acquired with a Bruker Avance Neo 600 MHz spectrometer (Bruker BioSpin, Karlsruhe, Germany) equipped with a Prodigy cryoprobe and using Topspin 4.0 software, applying a noesypr1D pulse sequence with spectral width 11 kHz, acquisition time 2.75 s, 64 scans, relaxation delay 4 s, and four dummy scans. All spectra were processed with an ACD NMR processor 12.1 (shortened to ACD, ACD Labs).

### Analysis of Spectral Signals

Each free induction decay (FID) was multiplied by a Lorentzian broadening of 0.3 Hz, before applying the Fourier transformation. Transformed spectra were further processed in ACD, performing manual phasing and automatic baseline correction *via* the FID reconstruction model. Peaks in the 1D-NMR spectra were initially identified by comparison with similar metabolomics characterizations reported in the literature (Tikunov et al., 2010; Aru et al., 2016; Digilio et al., 2016; Cappello et al., 2018). A more precise approach was then adopted, submitting known chemical shifts to public databases such as the Human Metabolome DataBase (Wishart et al., 2018) (HMDB), the Biological Magnetic Resonance DataBase (Ulrich et al., 2008) (BMRB), and nmrshiftdb2 (Kuhn and Schlörer, 2015), and checking the peak relative intensity, multiplicity, and J coupling for each proton of each identified compound. Uncertain assignments were confirmed with spiking experiments and by 2D-NMR spectra. Signal validation methods are reported in Supplementary file 3.

### Statistical Analysis

Peak counting and identification were performed on 10 randomly selected spectra for each matrix type (referred as “group” in this section; nine only in one case). Automatic peak picking was performed for each preprocessed spectrum in ACD, using the following parameters: *noise factor* = 2; *minimum signal to noise ratio* (S/N) = 3; *detect peak shoulders* enabled; and *peak type* = positive. The same preprocessed data were used for the UpSet plot: spectra were automatically bucketed to 944 intervals (*bucket width* = 0.01 ppm; *intelligent bucketing* enabled with 50% looseness; and *reference integration value* for the entire spectrum set to 1,000). Each bucket was group-averaged into a data matrix (y-axis: group names, x-axis: buckets), which was then converted into a binary data matrix by setting a specific threshold value of 3x S/N for each group. Spectral intervals containing only zeroes and false positives were discarded. The resulting data matrix, reduced to 442 buckets, was used to build an UpSet plot in R 4.0.3 with UpSetR library (Conway et al., 2017)**.** Statistical analysis was performed in R 4.0.3 with MetaboAnalystR 3.0 library (Pang et al., 2020) for all acquired hemolymph spectra (Figure 1, Supplementary file 1). Spectral NMR signals were manually bucketed, integrated, and normalized (see Table 1). The resulting raw data was Pareto scaled (Van den Berg et al., 2006) prior to the multivariate analysis. A principal component analysis (PCA) and a partial least square-discriminant analysis (PLS-DA) were performed to discover the features involved in group-specific variations, whereas the Shapiro–Wilk distribution test and Levene’s test were used to assess data distribution and the equality of variances, respectively. ANOVA and a T-test were applied to assess the statistical significance of parametric data (*p* = 0.05). Kruskal–Wallis and Dunn tests were used to assess the statistical significance of nonparametric data (*p* = 0.05). Signal variations were displayed by a fold change (threshold = 1.1 log_2_ FC). Correlation heatmaps of metabolites showing a significant variation in the immunostimulation trial were obtained from Pareto-scaled data, using Pearson’s correlation coefficient (r). Coefficients with *p* < 0.05 were considered significant.

**TABLE 1:** *Mytilus galloprovincialis* hemolymph: NMR signal assignments from 0 to 9 ppm. For each chemical shift (first column from left) the assigned metabolite, the relative functional group, the atom (carbon or nitrogen) number referring to the molecular position of protons, and multiplicity (s, *singlet; d, doublet; t, triplet; q, quartet;* m, *multiplet; and na,* not assigned) are specified. The “integrated peaks” column specifies the portion of the signal considered for integration (inside vertical bars). Putative assignments are indicated in parentheses. Unknown metabolites are numbered (Unknown two or U2, etc.). Signal validation methods are reported in Supplementary file 3.

Chemical shift (ppm)	Assignment	Reference functional group	Atom n°	Multiplicity	Integrated peaks
0.92–0.94	L-Isoleucine	CH_3_	C5	t	1:|2:1|
0.963	L-Leucine	CH_3_	C5	t	1:|2|:1
0.972	L-Leucine	CH_3_	C5	t	|1|:2:1
0.985	L-Valine	CH_3_	C4	d	1:|1|
0.997	L-Valine	CH_3_	C4	d	|1|:1
1.00–1.02	L-Isoleucine	CH_3_	C3	d	Whole
1.037	L-Valine	CH_3_	C3	d	1:|1|
1.049	L-Valine	CH_3_	C3	d	|1|:1
1.04–1.07	Propionic acid	CH_3_	C3	t	Whole
1.096	Mytilitol	CH_3_	C1	s	Whole
1.252	Unknown 2	na	na	s	Whole
1.32–1.35	L-Threonine	CH_3_	C4	d	1:1
1.47–1.497	L-Alanine	CH_3_	C3	d	1:1
1.497–1.52	Unknown 3	na	na	m	Whole
1.69–1.78	L-Lysine	CH_2_	C5	m	
	L-Leucine	CH_2_	C3	m	Whole
1.98–2.01	L-Proline	CH_2_	C4	m		
	L-Isoleucine	CH_2_	C3	m	Whole
2.03–2.04	Unknown 4	na	na	s	Whole
2.11–2.17	L-Glutamine	CH_2_	C3	m	
	L-Glutamic acid	CH_2_	C3	m	Whole
2.16–2.21	Propionic acid	CH_2_	C2	q	Whole
2.235	Acetone	CH_3_	C1,2	s	Whole
2.274	Acetoacetic acid	CH_3_	C4	s	Whole
2.33–2.36	L-Proline	CH_2_	C3	m	
	L-Glutamic acid	CH_2_	C4	m	Whole
2.41	Succinic acid	CH_2_	C2-3	s	Whole
2.43–2.48	L-Glutamine	CH_2_	C4	m	Whole
2.55–2.57	Beta-alanine	CH_2_	C2	t	1:2:1
2.65–2.67	Hypotaurine	CH_2_	C2	t	1:|2:1|
2.68–2.83	L-Aspartic acid	CH_2_	C3	m	Whole
2.86–2.88	L-Asparagine	CH_2_	C3	qd	11:22:22:|11|
2.91	Dimethylglycine	CH_3_	N	S	Whole
2.94–2.98	L-Asparagine	CH_2_	C3	qd	|11:22|:22:11
3.038–3.02	L-Lysine	CH_2_	C6	t	1:2:|1|
3.11	Unknown 5	na	na	s	Whole
3.135	(Malonic acid)	na	na	s	Whole
3.14–3.16	Unknown 6	na	na	s	Whole
3.233	Acetylcholine	CH_3_	N	s	Whole
3.21–3.243	L-Histidine	CH_2_	C3	m	1:|2:2:1|
3.27	Betaine	CH_3_	N	s	Whole
3.288	Taurine	CH_2_	C1	t	|1|:2:1
3.46–3.52	D-Glucose	CH	C2	m	Whole
3.57	*Glycine*	CH_2_	C1	s	Whole
3.71–3.82	CαAA	CH	na	na	Whole
3.91	Betaine	CH_2_	C2	s	Whole
3.962–3.997	L-Serine	CH_2_	C3	dd	1:1:|1:1|
4.18	(γ-Hydroxybutyric acid)	na	na	t	|1|:2:1
4.243–4.291	L-Threonine	CH	C4	m	Whole
4.37	Homarine	CH_3_	N	s	Whole
5.25	D-Glucose	CH	C1	d	Whole
6.15	ATP/ADP	na	na	m	Whole
6.91	L-Tyrosine	CH	C6	d	Whole
7.15	L-Histidine	CH	C5	d	Whole
7.19	L-Tyrosine	CH	C7	d	Whole
7.42–7.44	L-Phenylalanine	CH	C5;9/C6;8	m	Whole
7.56	Unknown 8	na	na	d	Whole
7.74	Unknown 9	na	na	d	Whole
8.45–8.46	Formic acid	CH	C1	s	Whole
8.53–8.57	Homarine	CH	C5	t	Whole

### Quantification of Hemolymph Metabolites

Twenty-eight known metabolites, detected in *Vibrio*-injected mussels at 12 and 24 h, have been quantified as follows: the spectra of all hemolymph samples obtained from immune-stimulated and mock-injected mussels were aligned with speaq 2.0 (Beirnaert et al., 2018) and deconvoluted with BATMAN (Hao et al., 2014). The metabolite concentration was determined by external calibration (Burton et al., 2005) using a calcium formiate standard solution (TraceCERT® Merck). 1D spectra of one of the hemolymph samples and of the standard solution were acquired under identical conditions using a long relaxation delay (120 s) to provide quantitative data of all the metabolites. In this way, the metabolite concentration of the reference sample was determined. The concentration of metabolites in all other samples was obtained by comparing the absolute areas of the metabolite signals with that of the reference sample on the spectra acquired with the acquisition parameter reported above (see paragraph *Tissue processing and acquisition of 1H 1D-NMR spectra*). The concentration is reported as µM (µmoles of metabolite per liter of hemolymph).

## Results

### Preliminary NMR Characterization of Mussel Hemolymph and Tissues

**FIGURE 2:**
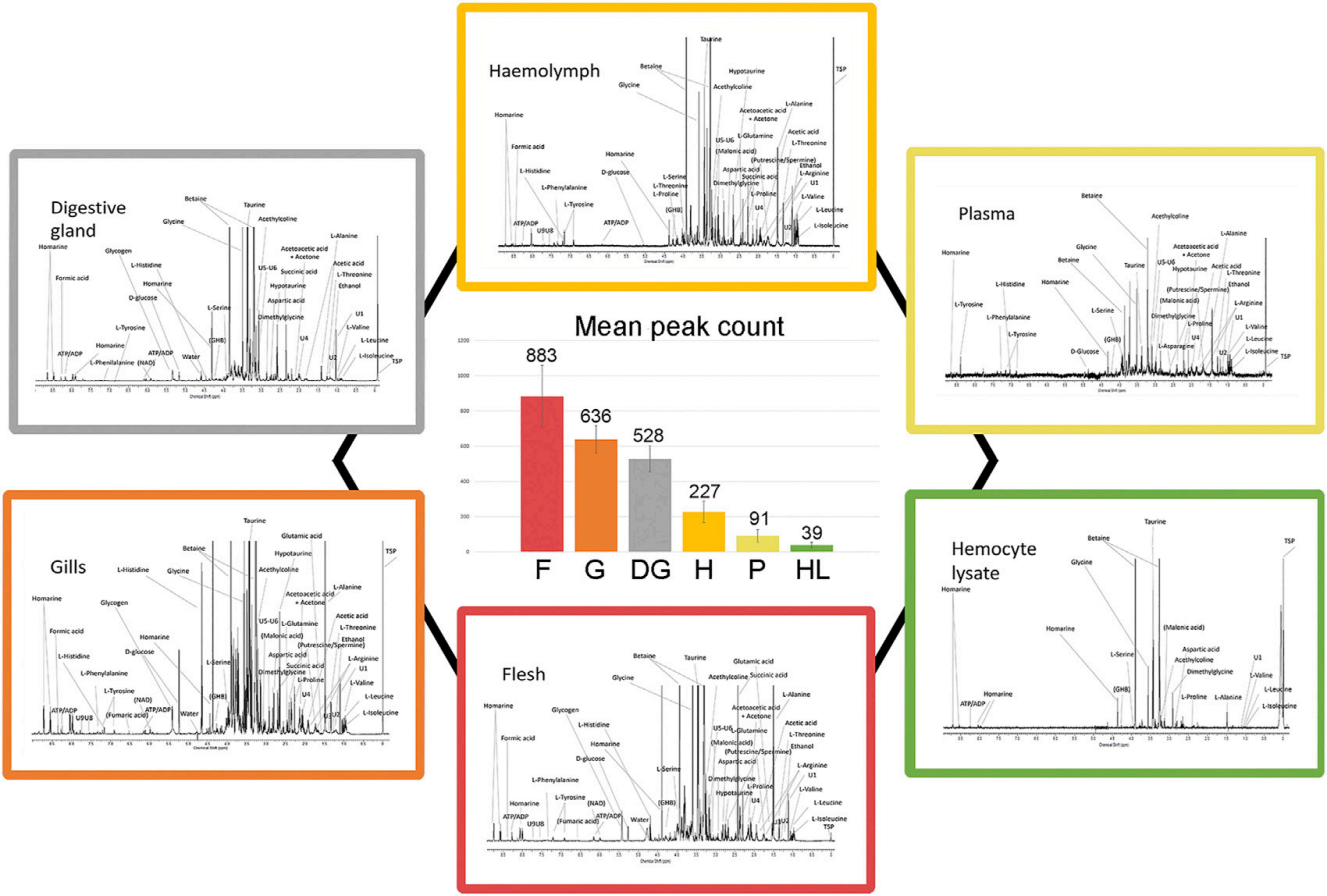
Typical ^1^H 1D-NMR profiles of different mussel matrices and relative signal abundance. Each peripheral profile includes the prominent signal assignments referred to polar extracts of mussel flesh, gills, and digestive gland as well as hemolymph, plasma, and hemocyte lysate (see Supplementary file 3–4 for details on the spectral scale and assignments). The six spectra are proposed at different signal intensity scales for better visualization. The central diagram indicates the number of spectral peaks, averaged on 10 spectra.

We analyzed aqueous samples (hemolymph, plasma, and hemocyte lysate) and polar tissue extracts (whole flesh, gills, and digestive gland) from sampled mussels acclimated three days in SW at 18°C. A total of 300 ^1^H 1D-NMR spectra were acquired to explore the metabolic profile of the analyzed biological matrices. Representative spectra are reported in Figure 2 (Supplementary file 3 and 4 for details). Whole flesh (F) proved to be the most complex tissue matrix, with almost 900 signals above a 3x S/N threshold. Clear differences in the number of tissue-specific metabolites were appreciable between gills (G) and digestive gland (DG), with the latter exhibiting fewer spectral peaks (636 ± 79 in gills, 528 ± 73 in digestive gland). Hemolymph (H), plasma (P), and particularly the hemocyte lysate (HL) showed far simpler profiles, with a maximum peak count of 227 in the first matrix (Figure 2).

**FIGURE 3:**
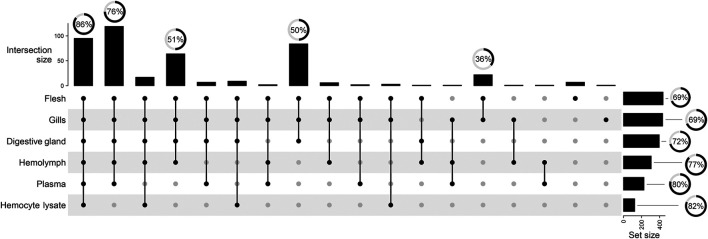
UpSet plot summarizing the grouping of total 442 spectral buckets across all mussel matrices. Vertical lines visualize the signal intersection between matrices. Black dots indicate the matrices involved in the intersection. Vertical bars show the size of each intersection, whereas horizontal bars indicate the number of spectral buckets above the 3x S/N threshold. Radial charts reported over bars depict the percentage of assigned spectrum signals.

Matrix-specific variations in total signal counts are illustrated in Figure 3. Polar tissue extracts retain most of the complexity in terms of signal number, with 19% of total signal buckets being unique to F, G, and DG. F and G further differentiate from DG, with 5% of signal buckets in common. Conversely, the aqueous samples showed fewer unique signals and 27% of signals in common with the polar extracts (see dot and line intersections in Figure 3). The spectral buckets common to all analyzed matrices show the highest percentage (86%) of assignments, whereas half of the buckets in common to all polar extracts remain unknown.

#### Common Spectral Features

Osmolytes showed a pervasive presence and high signal intensity in all analyzed matrices, highlighting the importance of osmoregulation in mussel physiology. Among them, taurine and hypotaurine are organosulfur compounds that only differ for the presence of a sulfonic or a sulfinic group, respectively. Moreover, N,N,N-trimethylglicine or betaine, trimethylamine N-oxide (TMAO), and homarine share a methylated quaternary ammonium, whose positive charge is counterbalanced by a carboxylic group in betaine and homarine and by an N-oxide functional group in TMAO. These chemical features underlie key biological functions, such as cellular water retention and methyl-donation (Petronini et al., 1992).

Most of the assigned signals derive from amino acids (AA). From right to left, the following signals were assigned: branched chain amino acid (BCAA) resonances were found in the shielded alkyl region, all with comparable intensity, along with the 7-C sugar alcohol mytilitol. The signal of the alanine methyl group was particularly intense. Glutamine, glutamic acid, arginine, and proline accounted for several partially overlapping multiplets between 1.5 and 2.5 parts per million (ppm). Glycine showed very high signal intensity, comparable to osmolytes. Sugar moiety resonances, mainly D-glucose, and Cα protons of amino acids appeared in a densely populated spectral region (3.5–3.90 ppm). Resonances from serine and threonine Cβ protons and from N-methyl groups of homarine and trigonelline were observed between the betaine methylene proton resonances (3.91 ppm) and the water signal (4.6–5.0 ppm, variable). In the aromatic region, we attributed the most shielded protons to tyrosine, followed by histidine, phenylalanine, homarine, and trigonelline (putative). Several metabolic products/intermediates, such as acetic acid, acetoacetic acid, ethanol, fumaric acid (putative), γ-hydroxybutyric acid (putative), malonic acid (putative), and succinic acid, were found in different portions of the spectrum. The anomeric protons of α-D-glucose resonate at 5.25 ppm; a broad signal centered at 5.41 ppm arises from the same protons in the polymer glycogen. At around 6 ppm, signals from the ribose component of ATP, ADP, and NADH were detected.

### Hemolymph Metabolite Changes in Mussel Acclimated at 18 and 25°C

**FIGURE 4:**
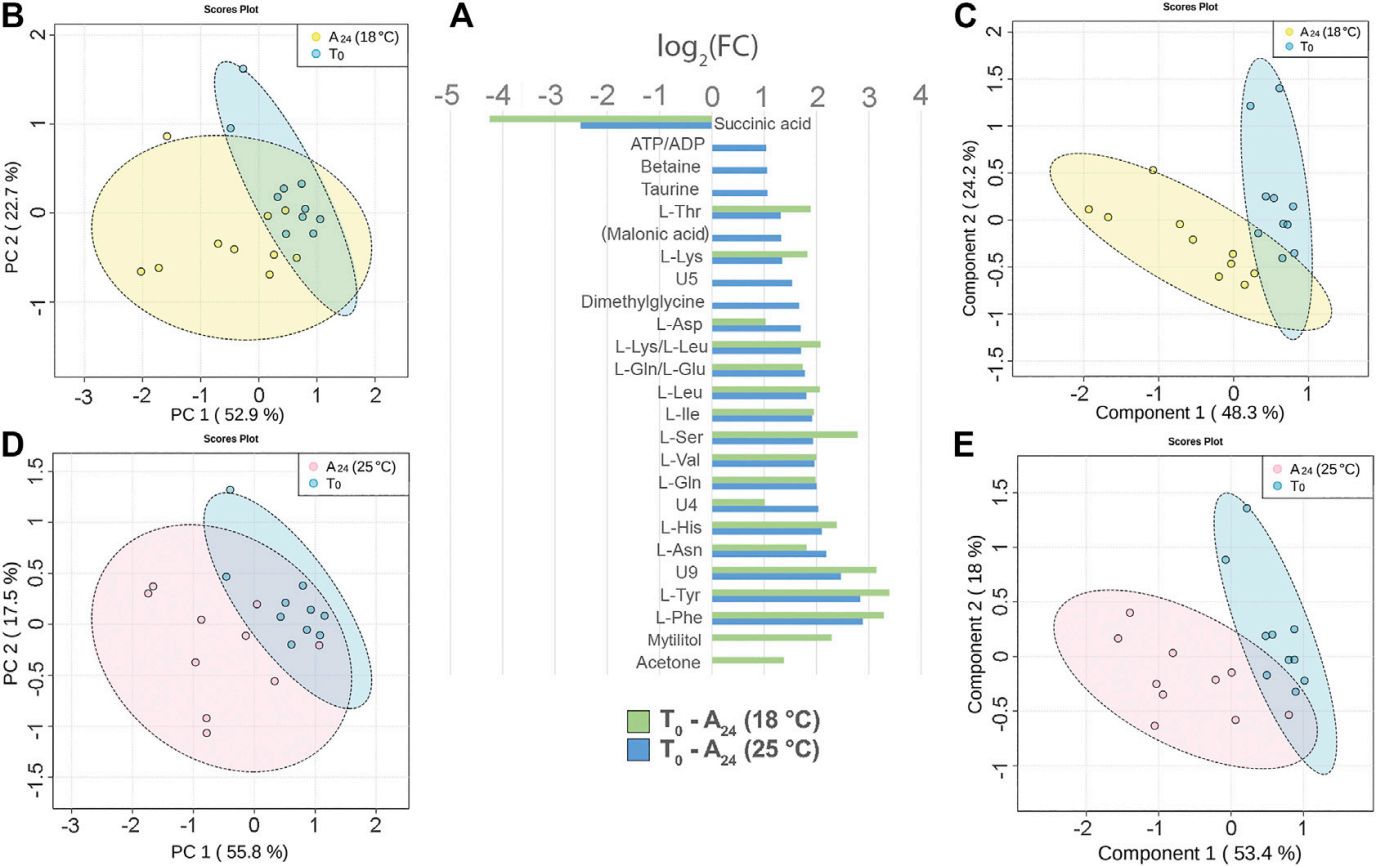
Hemolymph metabolite changes after three days of acclimation at 18°C and 25°C of mussels transported for 3 h in air. **(A)** Paired columns showing all metabolites significantly altered at 18°C (green) and 25°C (blue). The x-axis indicates the extent of metabolite variation in log_2_ fold change for the acclimated groups A_24_ (18°C) and A_24_ (25°C) related to the T_0_ group. The PCA **(B)** and the PLS-DA **(C)** based on NMR data of the acclimated A_24_ (18°C) and T_0_ mussels (depicted in yellow and blue, respectively). The PCA **(D)** and the PLS-DA **(E)** of the acclimated A_24_ (25°C) and T_0_ mussels (depicted in pink and blue, respectively). The scale values along the x- and y-axes of panels B, C, D, and E are divided by 10^6^ for a better visualization, whereas the colored ellipses indicate the 95% confidence region.

Three days acclimation of mussels (N = 10) at 18 or 25°C, after 3 h transport in air, altered the signal intensity of 19 and 23 hemolymph metabolites, respectively. The comparison of the ^1^H 1D-NMR spectra of the acclimated mussels, namely, A_24_ (18°C) and A_24_ (25°C), *versus* T_0_ mussels indicated a significant decrease of succinic acid and an increased intensity of most free AA both at 18°C and at 25°C (Figure 4A). The free AA in common at the two acclimation temperatures exhibited a rather similar variation profile, with a mean increase of 2.15 log_2_ fold change (18°C) and 1.96 log_2_ fold change (25°C) relative to T_0_. Conversely, mytilitol and the ketone body acetone showed an increase only at 18°C, whereas osmolytes increased, to smaller degree, only at 25°C. In two out of 10 hemolymph spectra from T_0_ mussels, we also identified the signals of propionic acid.

The principal component analysis (PCA) made evident the cluster of T_0_ hemolymph samples, whereas the hemolymph samples from both acclimation temperatures were more scattered.

At 18°C, the PC1 and PC2 axes explained 52.9 and 22.7% of the total dataset variance (Figure 4B). The partial least square discriminant analysis (PLS-DA) produced a more defined separation of the two sample groups (Figure 4C). The cross validation of the model indicates a good prediction capability with an accuracy of 0.95, a multiple correlation coefficient (R^2^) of 0.8, and a cross-validated R^2^ (Q^2^) of 0.66 (three-component model, Supplementary file 5). Nine metabolites contributed the most to the sample distribution in PLS-DA, as indicated by their variable importance in projection (VIP) scores (VIP > 1.0).

At 25°C, the PC1 and PC2 axes explained 55.8 and 17.5% of the total dataset variance, respectively (Figure 4D). PLS-DA produced a good separation of the two sample groups (Figure 4E). Cross validation of the model indicates a good prediction capability with accuracy = 0.95, R^2^ = 0.82, and Q^2^ = 0.56 (three-component model, Supplementary file 5). Six metabolites contributed the most to the sample distribution in PLS-DA, as indicated by their VIP scores (VIP >1.0).

Concerning the effect of the temperature on mussel acclimation, the mussels at 18°C display a significantly higher level of ketone body acetone and a lower level of succinic acid than the 25°C group.

### Hemolymph Metabolite Changes at 12 and 24 h After *Vibrio* Injection (18°C)

**FIGURE 5:**
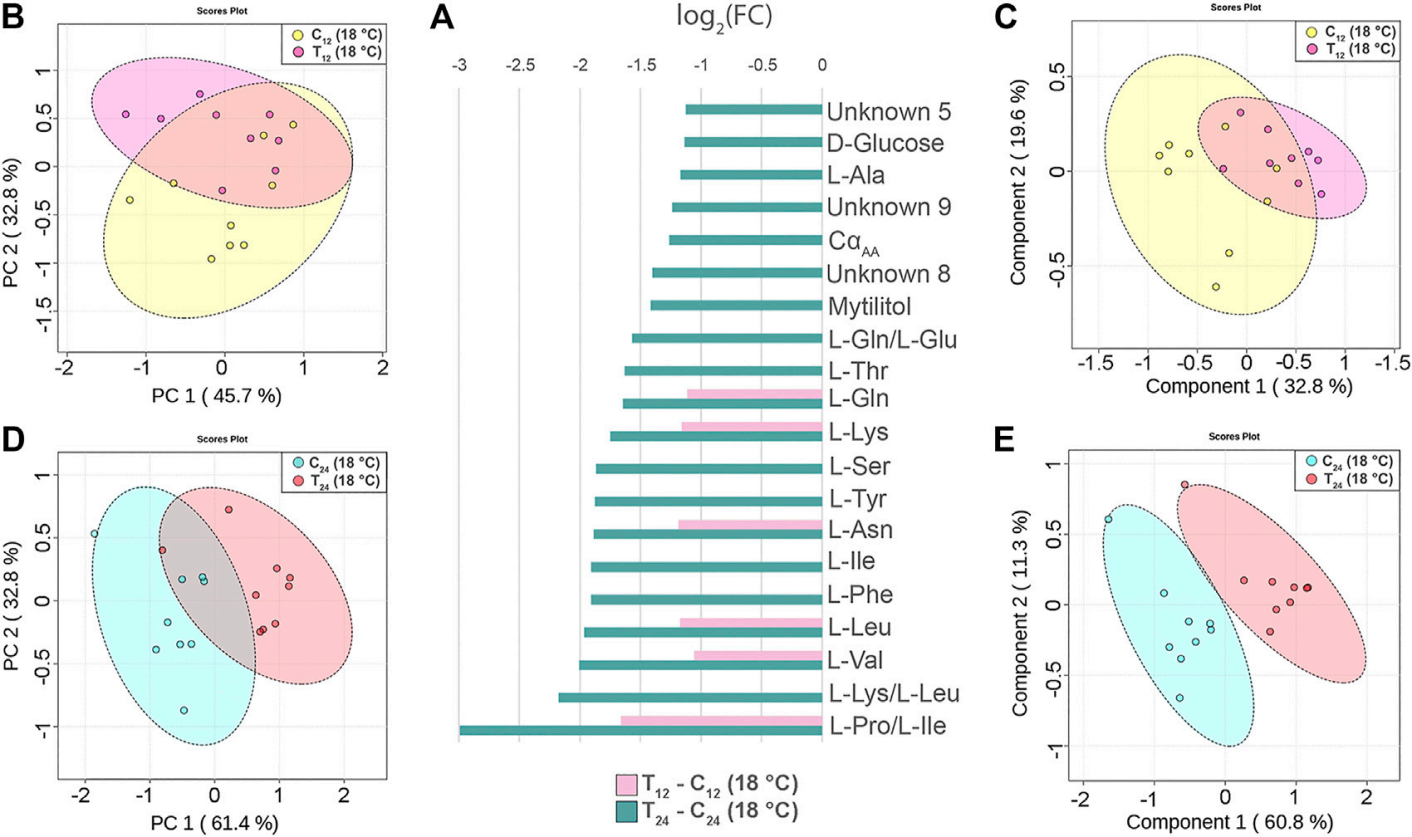
Hemolymph metabolite changes in *Vibrio*-injected mussels at 12 and 24 h postinjection (hpi). **(A)** Paired columns showing all metabolites significantly altered at 12 hpi (pink) and 24 hpi (green) in T_12_ and T_24_ mussels. The x-axis indicates the extent of metabolite variation in log_2_ fold change related to C_12_ and C_24_ time-paired controls. The PCA **(B)** and the PLS-DA **(C)** based on NMR data of the *Vibrio*-injected mussels T_12_ and control mussels C_12_ (depicted in pink and yellow, respectively). The PCA **(D)** and the PLS-DA **(E)** of the *Vibrio*-injected mussels T_24_ and control mussels C_24_ (depicted in red and light blue, respectively). The scale values along the x- and y-axes of panels B, C, D, and E are divided by 10^6^ for a better visualization, whereas the colored ellipses indicate the 95% confidence region.

The injection of 2x10^7^ CFU of *V. splendidus* in mussels maintained at 18°C caused a generalized reduction of the signal intensity at either 12 or 24 hpi (N = 9, time-paired controls). The mussel response at 12 hpi was characterized by signal reduction of six amino acids (Figure 5A). Treated and control groups are not well separated in both the PCA and PLS-DA models (Figures 5B,C). Indeed, cross-validation of the PLS-DA model indicates low prediction capability of the model (accuracy = 0.77, R^2^ = 0.65, and Q^2^ = 0.3, three-component model, Supplementary file 5).

Conversely, at 24 hpi, the *Vibro-*treated mussels showed a significant reduction of 20 signals attributable to 13 different amino acids, two different sugars, and three unknown compounds (Figure 5A). The PCA and PLS-DA models display a clear-cut separation between the control and treated groups (Figures 5D,E). The PC1 and PC2 axes of PCA explain 61.4 and 32.8% of the total dataset variance, respectively, whereas PLS-DA shows a strong predictive capability (accuracy = 0.94, R^2^ = 0.94, and Q^2^ = 0.7, four-component model, Supplementary file 5). Twelve metabolites contributed the most to the sample distribution in PLS-DA, as indicated by their VIP scores (VIP > 1.0). Signal quantification is reported with related standard error in Supplementary file 7.

### Correlation Heatmaps (*Vibrio*-Injected vs. Mock-Injected Mussels)

**FIGURE 6:**
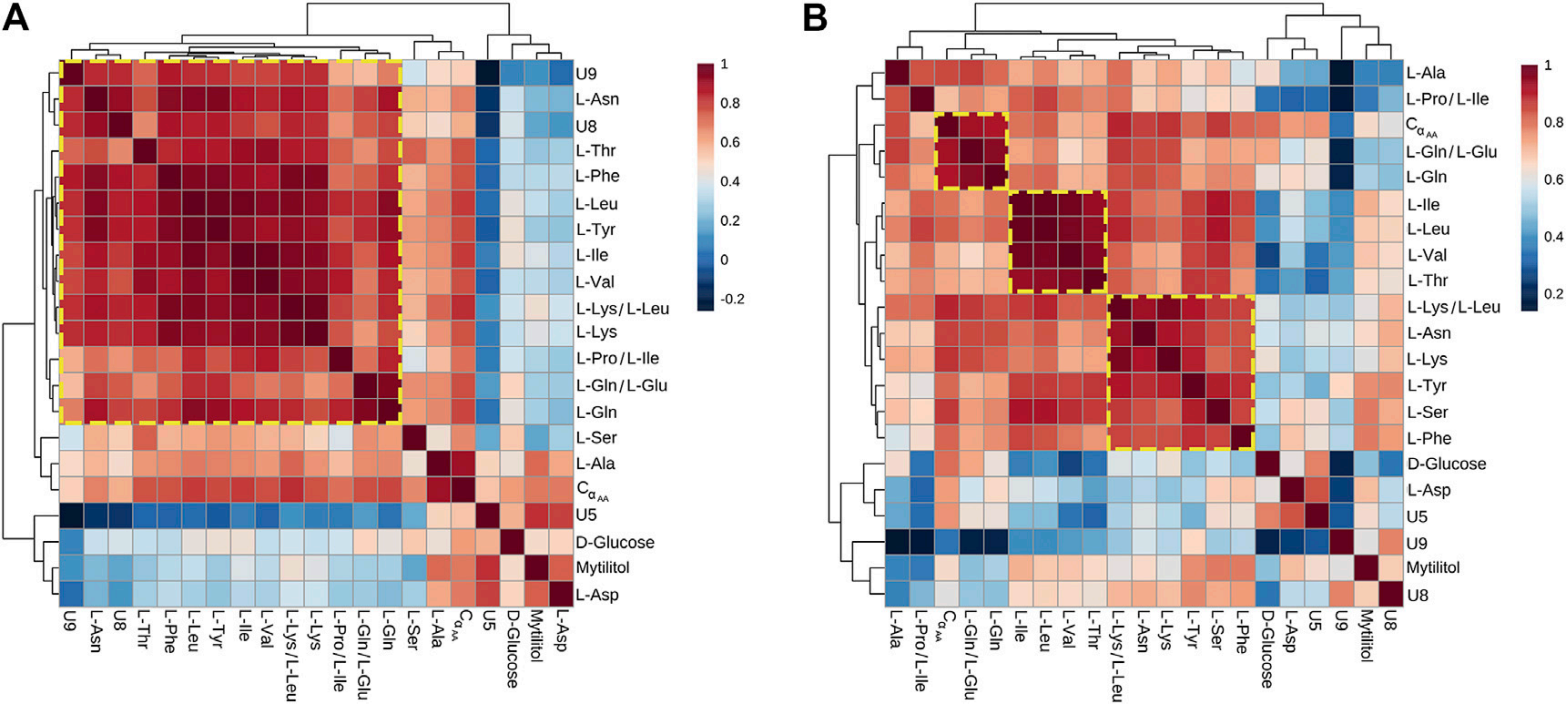
Correlation heatmaps of Pareto-scaled NMR data obtained from hemolymph of mussel mock- and *Vibrio*-injected, sampled at 12 **(A)** and 24 **(B)** hpi. The color scale refers to pair-wise Pearson's correlation coefficients and ranges from a maximum of 1 (red) to a minimum of −0.2 at 12 hpi and 0.2 at 24 hpi (dark blue). See *p*-values in Supplementary file 6.

All metabolites found significantly altered in the hemolymph of *Vibrio*-injected vs. mock-injected mussels at 24 hpi were used to build metabolic correlation maps, which are expected to group together compounds showing similar variation patterns and, therefore, to uncover stimulus-specific relationships between metabolites (Figure 6). At 12 hpi, the correlation analysis roughly outlined one single cluster having a great internal complexity.

At 24 hpi, three clusters were observed: the first one includes L-Ile, L-Leu, L-Val, and L-Thr; the second one is composed by L-Phe, L-Tyr, L-Lys, L-Asn, and L-Ser; and the third one is composed by L-Gln and L-Glu.

### Flow Cytometry and Sorting of Hemocytes From Immunostimulated Mussels

**FIGURE 7:**
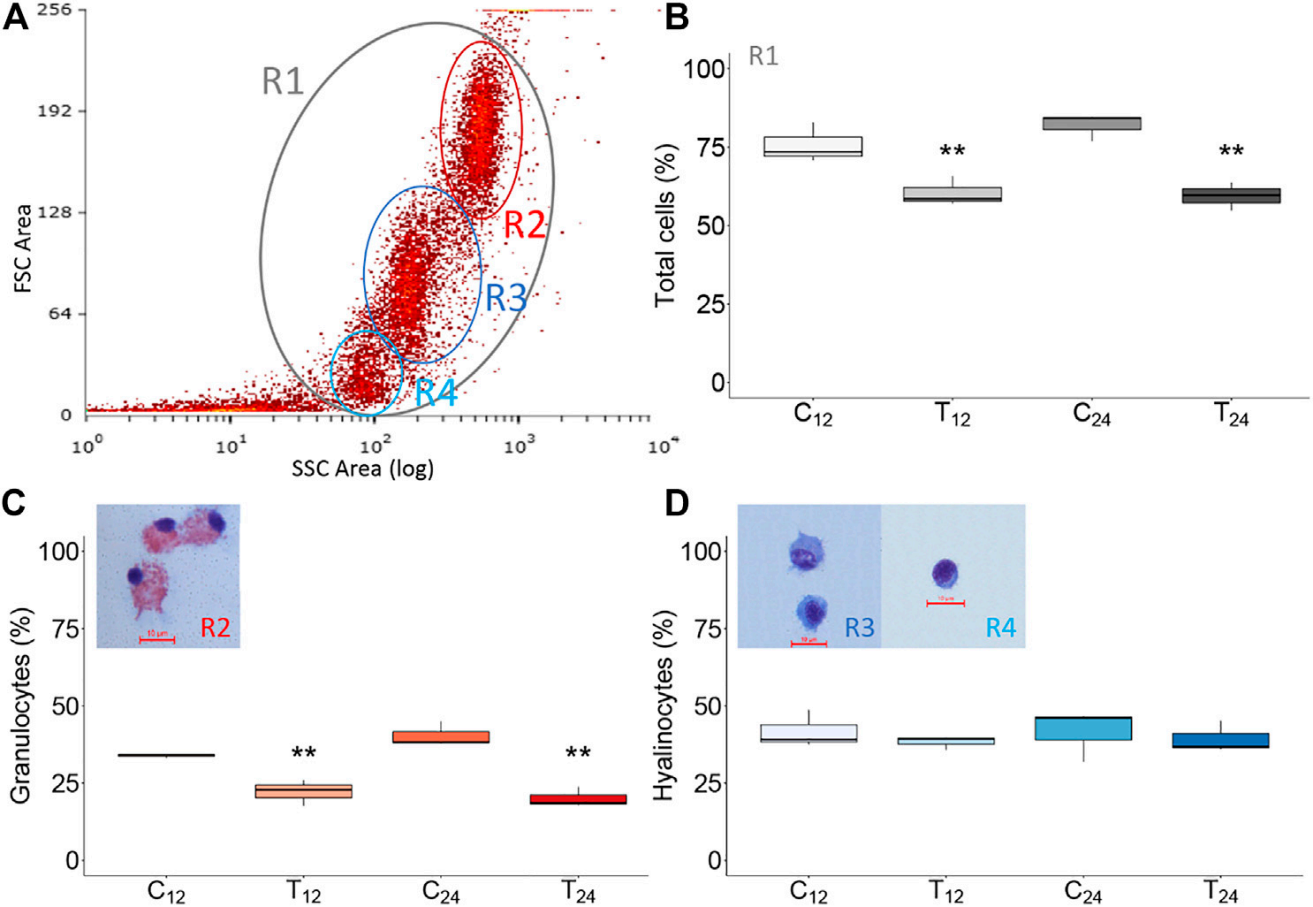
Flow cytometry analysis of mussel hemocytes from mock-injected (C_12_, C_24_) and *Vibrio*-injected (T_12_, T_24_) mussels. **(A)** Representative dot plot of hemocytes from control mussels (10,000 events; R2, granulocytes; R3, hyalinocytes, and R4 small hyalinocytes). **(B–D)** Graphical representation (box plot) of sorted hemocyte subpopulations in different experimental conditions (see text). Asterisks indicate the statistical significance: **p < 0.01. Naming of the experimental mussel groups are specified in Figure 1.

The hemocytes of the same mussels injected with filter-sterilized SW or *V. splendidus* were analyzed in flow cytometry to ascertain total hemocyte counts and the relative abundance of hemocyte subpopulations. Based on the forward scatter (FSC) and the side scatter (SSC) values, we clearly identified the two main subpopulations of mussel hemocytes (Figure 7). The first one was characterized by a greater internal complexity and size (R2 region, granulocytes), whereas the second one exhibited a lower internal complexity and smaller size (R3 region, hyalinocytes). A third group of cells was present in some samples and displayed the same internal complexity as the R3 subpopulation but smaller size (R4 region). After cell sorting, the identity of the different hemocyte subpopulations was confirmed by cytological analysis, with the R2 and R3 subpopulations representing granulocytes and hyalinocytes, respectively. The R4 subpopulation (when present) mostly contained small hyalinocytes but also contained small cells with a high nucleus/cytoplasm ratio. Flow cytometry and cell sorting were used to trace the variation in the number of total hemocytes and the abundance of hyalinocytes and granulocytes among the differently challenged mussels. At 12 hpi, the total number of hemocytes significantly decreased (*p*-value 0.002) in the paired comparison of the control and the treated groups, and such a difference was even more significant at 24 hpi. Concerning the hemocyte population structure, the fraction of hyalinocytes did not show any significant change during the immunostimulation trial. Conversely, the fraction of granulocytes significantly decreased after injection of live *V. splendidus*, at both 12 and 24 hpi, from 33.1 to 22.1% and from 40.3 to 20.0%, respectively (Figure 7).

## Discussion

We acquired ^1^H 1D-NMR spectra of *Mytilus galloprovincialis* hemolymph to investigate the metabolic processes of mussels acclimated in seawater at 18 and 25°C, after 3 h-transport out of water, and mussels immunostimulated at 18°C by injection of 2 × 10^7^ CFU of *Vibrio splendidus*.

Primarily, we explored the metabolite profiles of hemolymph, plasma, and hemocyte lysate (aqueous matrices) as well as whole flesh, digestive gland, and gills (polar tissue extracts) of mussels acclimated at 18°C. The NMR spectra of aqueous samples displayed lower complexity than those of polar extracts, the latter requiring careful signal assignment and deconvolution. We selected hemolymph as the matrix of choice because of its central role in the organism immunity and the possibility to acquire it “as is” with minor adjustments (see tissue processing and acquisition of ^1^H 1D-NMR spectra). Although hemolymph samples showed simpler NMR profiles, the spectra were quite crowded. We could identify a total of 61 resonances of 34 defined chemical species, plus seven unassigned signals. Four of these unknown (U) resonances are also reported in other studies: U2 and U5 [1.25 ppm, 3.11 ppm, (Watanabe et al., 2015)]; U6 and U9 [3.15 ppm, 7.74 ppm, (Tikunov et al., 2010; Digilio et al., 2016)]. Altogether, these findings are consistent with previous studies, apart from the occasional appearance of propionic acid (1.06 ppm, 2.06 ppm) in T_0_ hemolymph samples. Remarkably, Unknown 1 (Aru et al., 2016; Cappello et al., 2018) was recently identified as C-methyl-scyllo-inositol (mytilitol), a 7-C sugar alcohol proposed as a species-specific indicator of geographic origin (Aru et al., 2020).

The hemolymph NMR spectra from mussels acclimated at 18 and 25°C, relative to those of T_0_ mussels, showed significant changes in the levels of succinate, free AA and osmolytes, mytilitol, and some unknown metabolites (Figure 4). Succinate had the most relevant variation, with −4.25 and −2.51 log_2_ fold change (about 19 and 6 times less than T_0_ mussels) at 18 and 25°C, respectively. Overall, these changes likely reflect effective osmoregulation and the restart of aerobic respiration after 3 h of functional anaerobiosis.

Although mussels with closed valves preserve seawater in their palleal cavity, gas exchange is very limited and O_2_ is quickly depleted (Nicastro et al., 2010). The significant decrease of succinate in the acclimated mussels suggests its recruitment in the tricarboxylic acid cycle (TCA), following the accumulation of this intermediate during transient functional anaerobiosis. Annelida, Cnidaria, and Mollusca cope with anoxic/hypoxic conditions by performing glycolytic fermentation: the metabolic transition is initiated by the formation of oxaloacetate (OXA) from phosphoenolpyruvate (PEP) by the action of PEP-carboxykinase, an enzyme generally found in euryoxic invertebrate muscles, as opposed to vertebrate muscles (De Zwaan and Wijsman, 1976; Mustafa et al., 1983). Cytosolic OXA is reduced to malate by malate dehydrogenase upon entering the mitochondrion (thus, in the TCA cycle) and ultimately converted into succinate *via* fumarate. Succinate accounts for the largest part of end-products, together with the volatile acetate and propionate during functional anaerobiosis (De Zwaan and Mathieu, 1992). An increase in succinate levels can also be explained by the anaerobiosis-driven shunt of aspartate and alanine within the glucose–alanine and aspartate–succinate pathways, as proposed by Collicutt and Hochachka (1977). A clear-cut increase in succinate (and malonate) has been recently reported in *Perna canaliculus* mussels by GC/MS analysis after 3 h transport in air (Venter et al., 2021).

The spectral signals of carbohydrates did not significantly change in the hemolymph of acclimated mussels, apart from mytilitol, which has been hypothesized to play a role as a source of energy (Aru et al., 2020). The increased levels of free AA in the hemolymph of acclimated mussels support the return to the aerobic metabolism after functional anaerobiosis. According to previous findings (Zurburg and Kluytmans, 1980), the anoxia-driven accumulation of organic acids, such as succinate, acetate, and propionate, would mobilize Ca^2+^ from the bivalve shell. If so, the cellular uptake of free AA would compensate the Ca^2+^-related increase of hemolymph osmolarity, thus maintaining a balance between intracellular and extracellular fluids (Zurburg and Kluytmans, 1980). However, this hypothesis deserves further investigation.

The response of *M. galloprovincialis* to *V. splendidus* LGP32 has been thoroughly characterized in terms of transcriptomics and cellular dynamics. The mussel response to *Vibrio* bacteria consists in a rapid activation of hemocytes through interconnected intracellular signaling pathways, eventually leading to the expression of genes for antimicrobial peptides and other molecular effectors (Gerdol et al., 2018). Also, genes involved in the aminoacyl-tRNA biosynthesis such as phenylalanine–tRNA ligase (Rey-Campos et al., 2019b), glutamate synthase, transketolase (tktA), isocitrate dehydrogenase (icd), and leucyl-tRNA synthetase (leuS) were found altered in mud crabs infected by *Vibrio parahaemolyticus* (Kong et al., 2020). The hemolymph NMR spectra obtained from *Vibrio*-injected mussels vs. time-paired controls showed a decrease of six and 20 chemical moieties at 12 and 24 hpi, respectively, attributable to free AA, sugars, and unknown compounds. The *Vibrio*-induced host response at 12 hpi was less marked and produced a weaker separation of the control and treated mussels than at 24 hpi; this may reflect the gradual onset of an innate immune reaction, which is detectable already at two hpi (Tanguy et al., 2018) and translates in massive transcriptome changes at 24–48 hpi (Venier et al., 2011; Rey-Campos et al., 2019a).

Correlation heatmaps of the NMR data obtained from the immunostimulation trial highlighted clusters of more tightly correlated AA, especially at 24 hpi, altogether suggestive of dynamic metabolic adjustments. Indeed, the correlations observed in metabolomics data reflect all the reactions and regulatory processes occurring in a biochemical network (Rosato et al., 2018), and metabolic adjustments are fundamental when combating infection, tissue damage, and disease onset. The weak clustering observed at 12 hpi suggests a nonselective cellular uptake of free AA, attributable to enhanced degradation and synthesis of cellular proteins in response to stress, that is, maintenance metabolism (Hawkins, 1991). On the other hand, the clusterization observed at 24 hpi suggests a more selective and regulated AA utilization. Faster protein turnover might enhance mussel performance by facilitating the mobilization and selective redistribution or catabolism of amino acids, and the elimination of dysfunctional proteins (Hawkins, 1991; Ross et al., 2020).

The injection of *Vibrio* cells has been associated to significant changes in hemocyte adhesion, reduced lysosomal membrane stability, consequent enzyme release, and extracellular ROS and NO production (Balbi et al., 2013). Mussel hemocytes can clear 10^7^ live cells of *V. splendidus* within 24 h in SW at 20°C, with the total circulating cells sharply decreasing in a first phase postinjection and substantial changes in the hemocyte subpopulation structure (Parisi et al., 2008; Parisi et al., 2019). After the bacterial injection, without shell filing, we recorded by flow cytometry a significant and progressive decrease of total hemocytes, specifically granulocytes, at 12 and 24 hpi, in agreement to another recent study performed on *V. splendidus*–injected mussels with SW at 15°C (Rey-Campos et al., 2019a). The observed change is consistent with hemocyte degranulation and migration to the site of injection. In fact, granulocytes store in their cytosolic granules a variety of immune effectors, such as antimicrobial peptides and hydrolytic enzymes, and release them in response to several stimuli (Balseiro et al., 2011). As a matter of fact, flow cytometry and cell sorting to bivalve hemocytes can help understanding the dynamics of hemocyte subpopulations upon different experimental conditions despite the current lack of subpopulation-specific markers.

## Conclusion

This study highlights the dynamic nature of mussel hemolymph metabolites and, therefore, the importance of a rigorous approach to the study of biochemical processes in mussels and other bivalves. Among the possible factors of variability, animal transport and sample preprocessing can induce significant spectral changes and consequently affect data interpretation. The results achieved in the Mediterranean mussel, *Mytilus galloprovincialis*, support data recently obtained in the Greenshell mussel, *Perna canaliculus* (Venter et al., 2021). Moreover, the analysis of minimally processed hemolymph samples is advantageous as it allows the detection of low molecular weight volatiles and peculiar compounds, such as propionic acid and mytilitol. Relying on existing databases and published papers, this study further supports a wider and practical use of NMR-based metabolomics as an investigation tool integrative of other omics in bivalve mollusc research.

## Data Availability

The raw data supporting the conclusions of this article will be made available by the authors, without undue reservation.
