# Supplementary material for: NMR Metabolite Profiles of the Bivalve Mollusc Mytilus galloprovincialis Before and After Immune Stimulation With Vibrio splendidus
Source: Front Mol Biosci. 2021 Sep 3;8:686770. doi: 10.3389/fmolb.2021.686770 (PMC8447493; doi:10.3389/fmolb.2021.686770)

Supplementary file 2. Hemolymph bacterial counts from acclimated, mock-injected, and Vibrio-injected mussels at 18 °C.

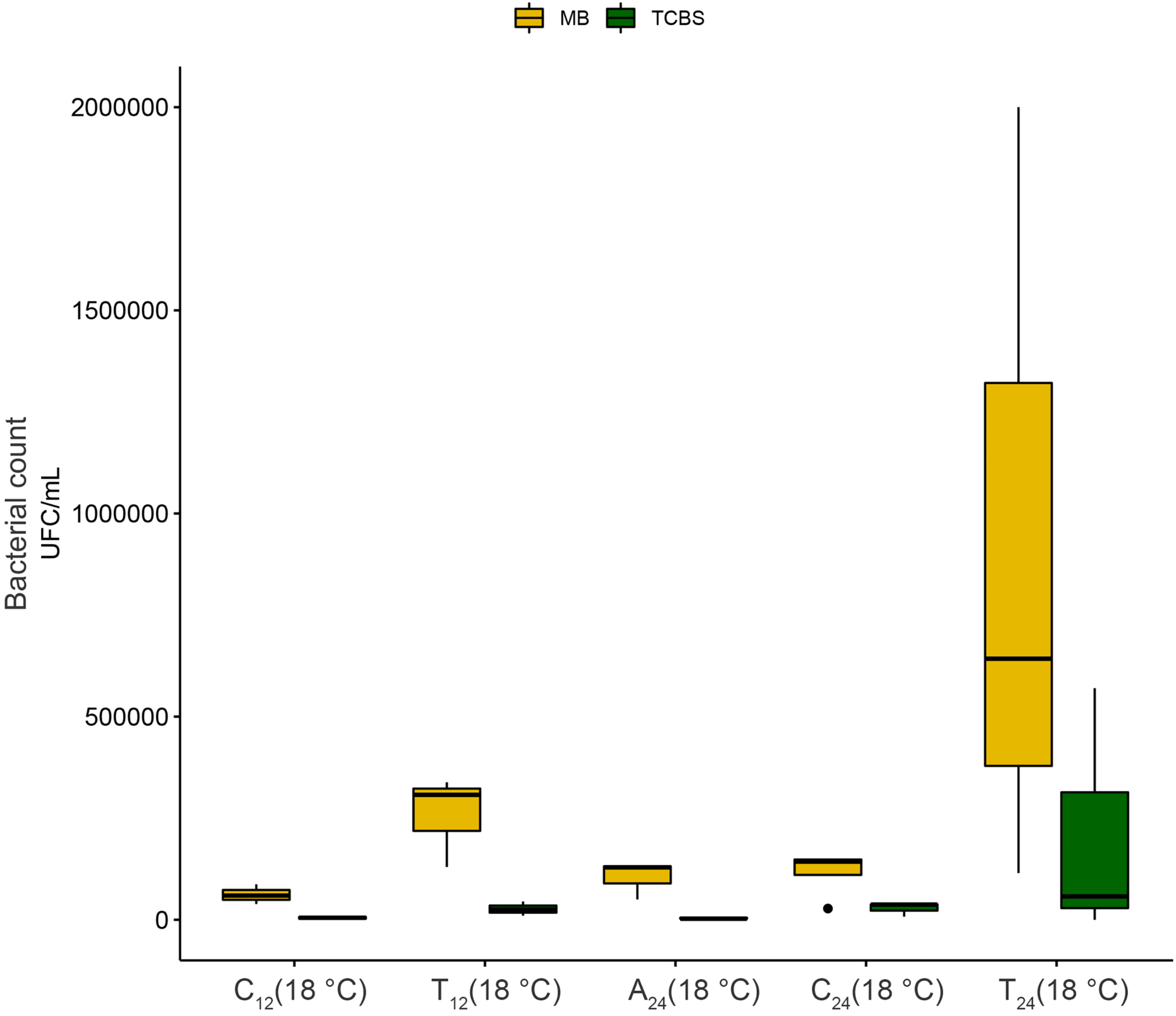

Supplement: Supplementary file 1 [file DataSheet2.pdf]
