# Supplementary figures and images for: NMR Metabolite Profiles of the Bivalve Mollusc Mytilus galloprovincialis Before and After Immune Stimulation With Vibrio splendidus
Source: Front Mol Biosci. 2021 Sep 3;8:686770. doi: 10.3389/fmolb.2021.686770 (PMC8447493; doi:10.3389/fmolb.2021.686770)

# Supplementary file 4. Typical <sup>1</sup>H 1D-NMR profiles of different mussel matrices

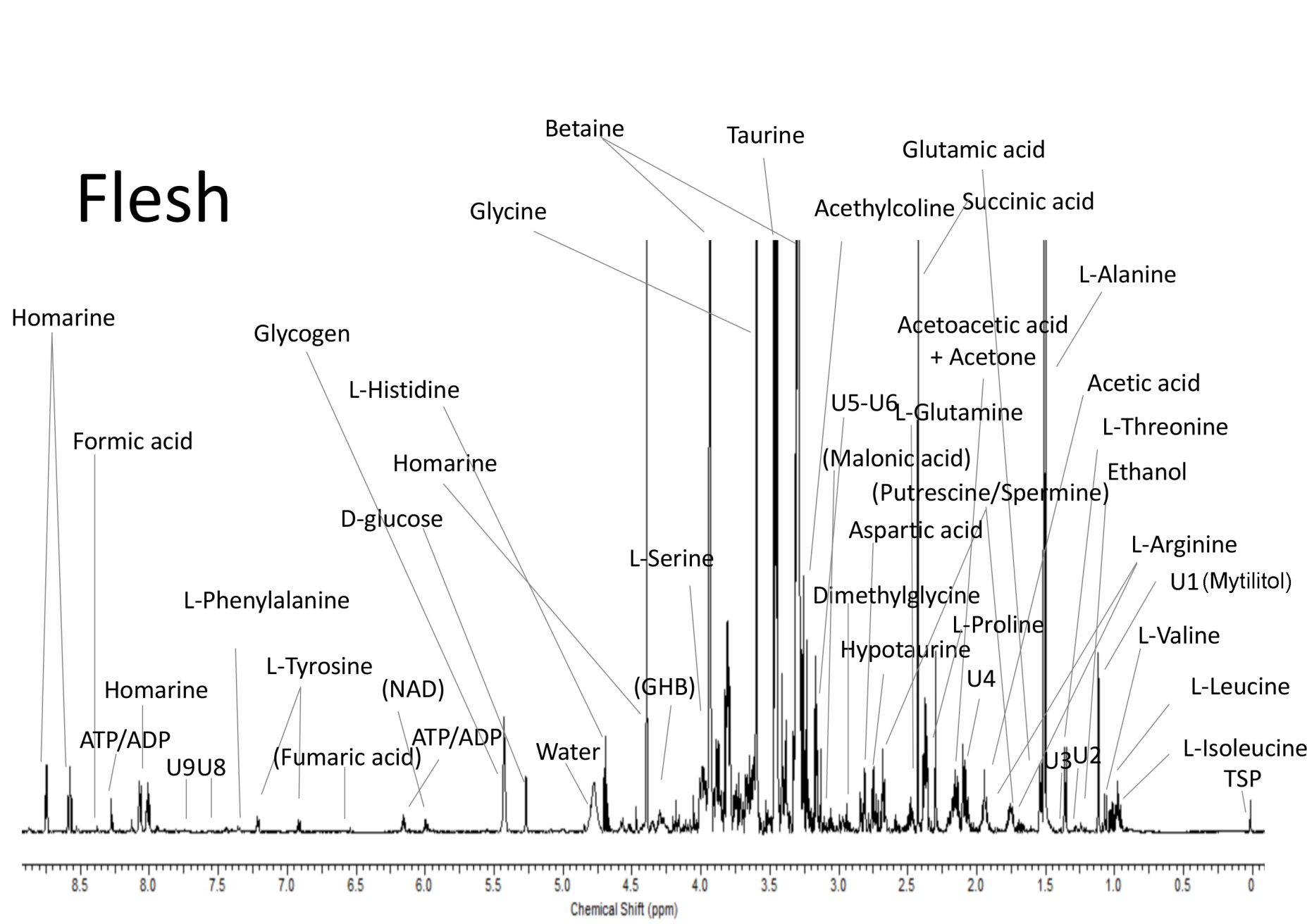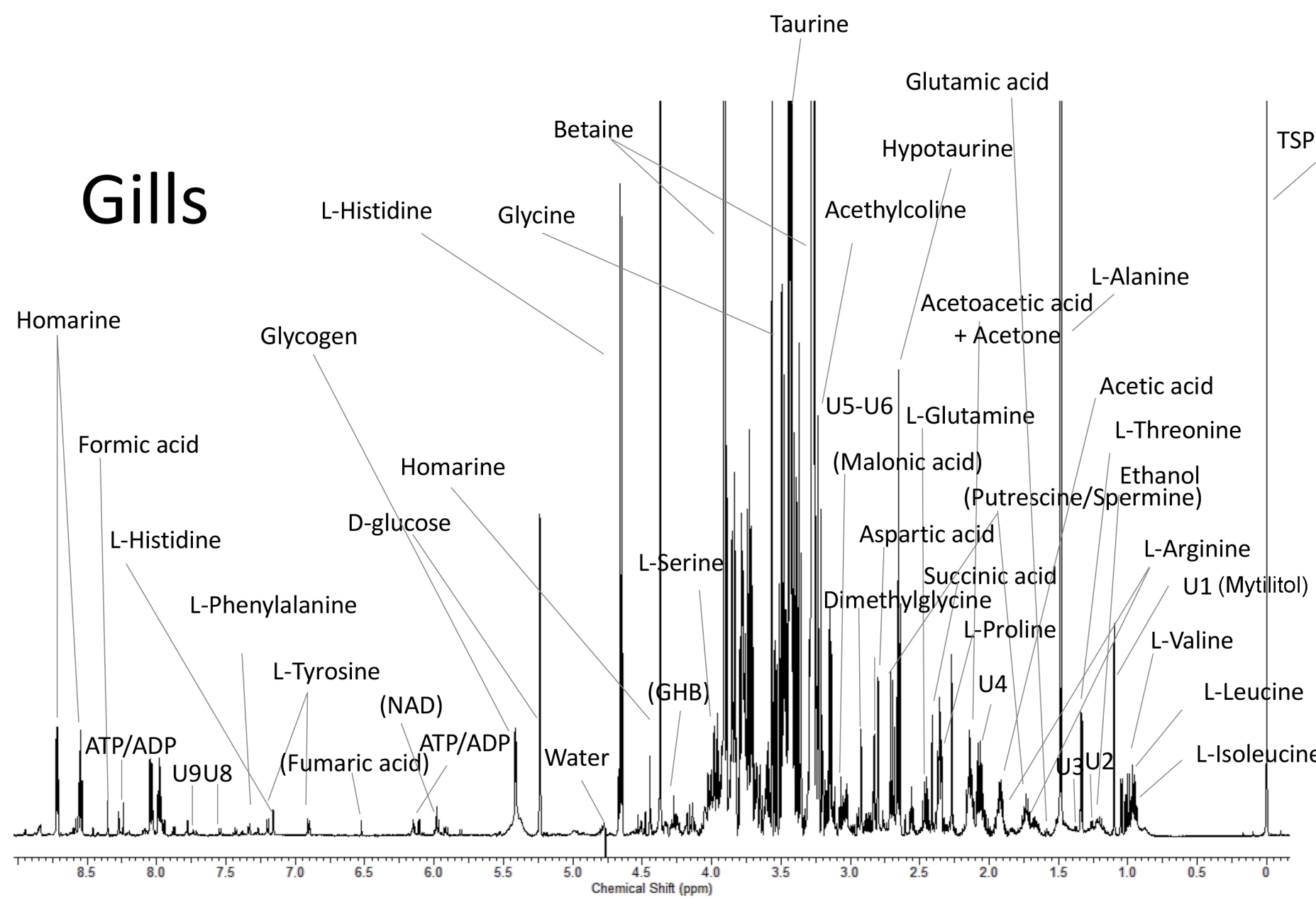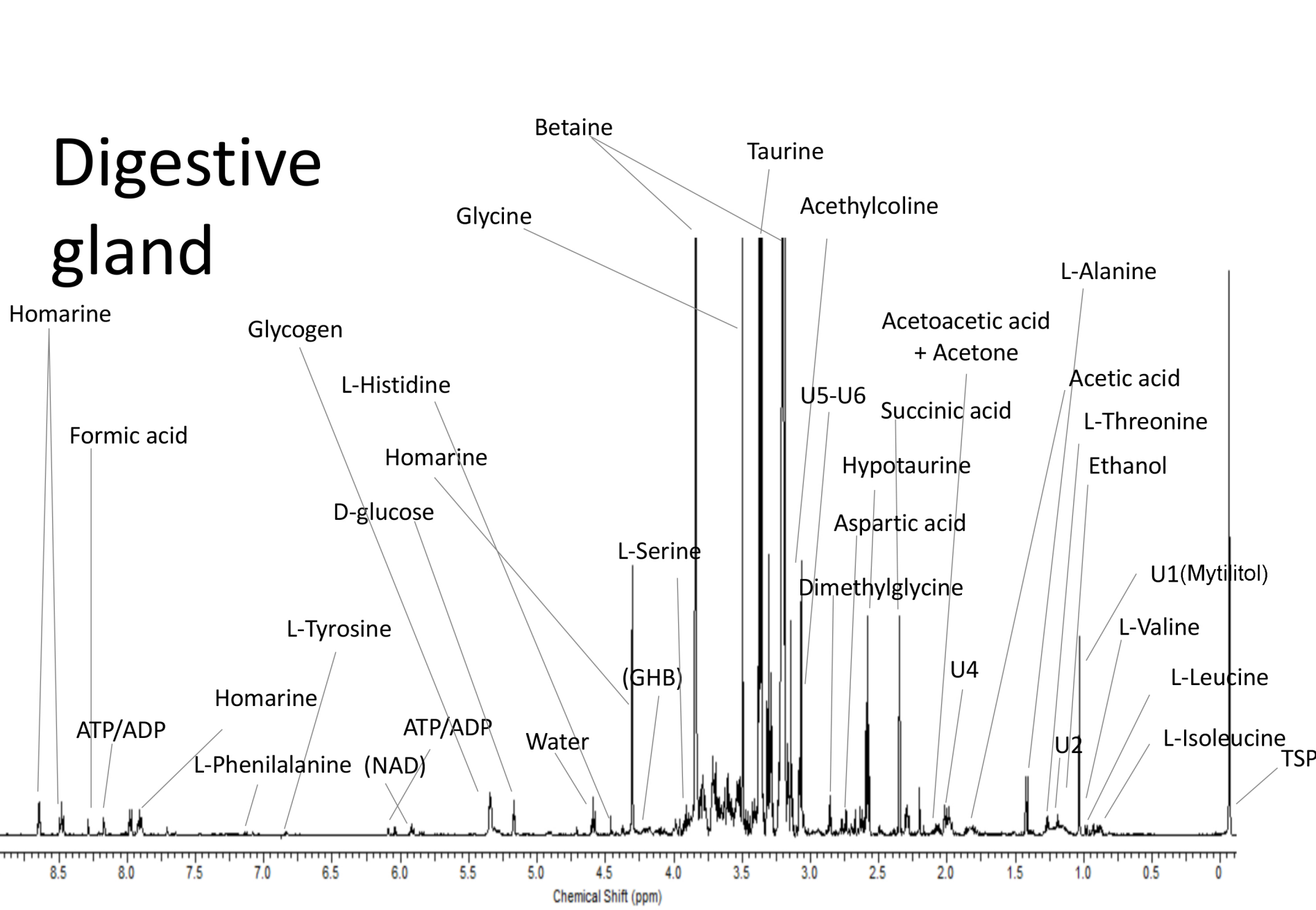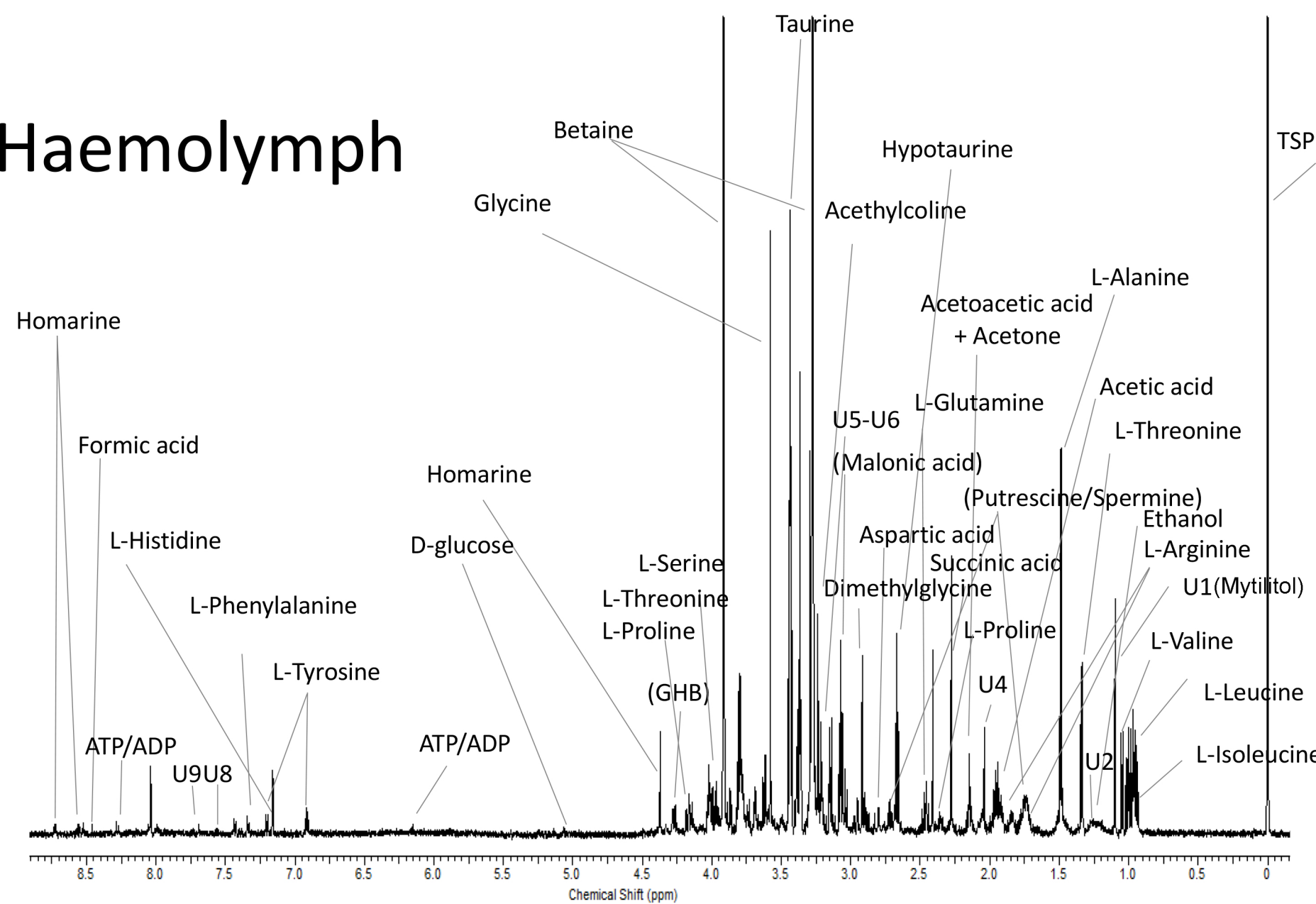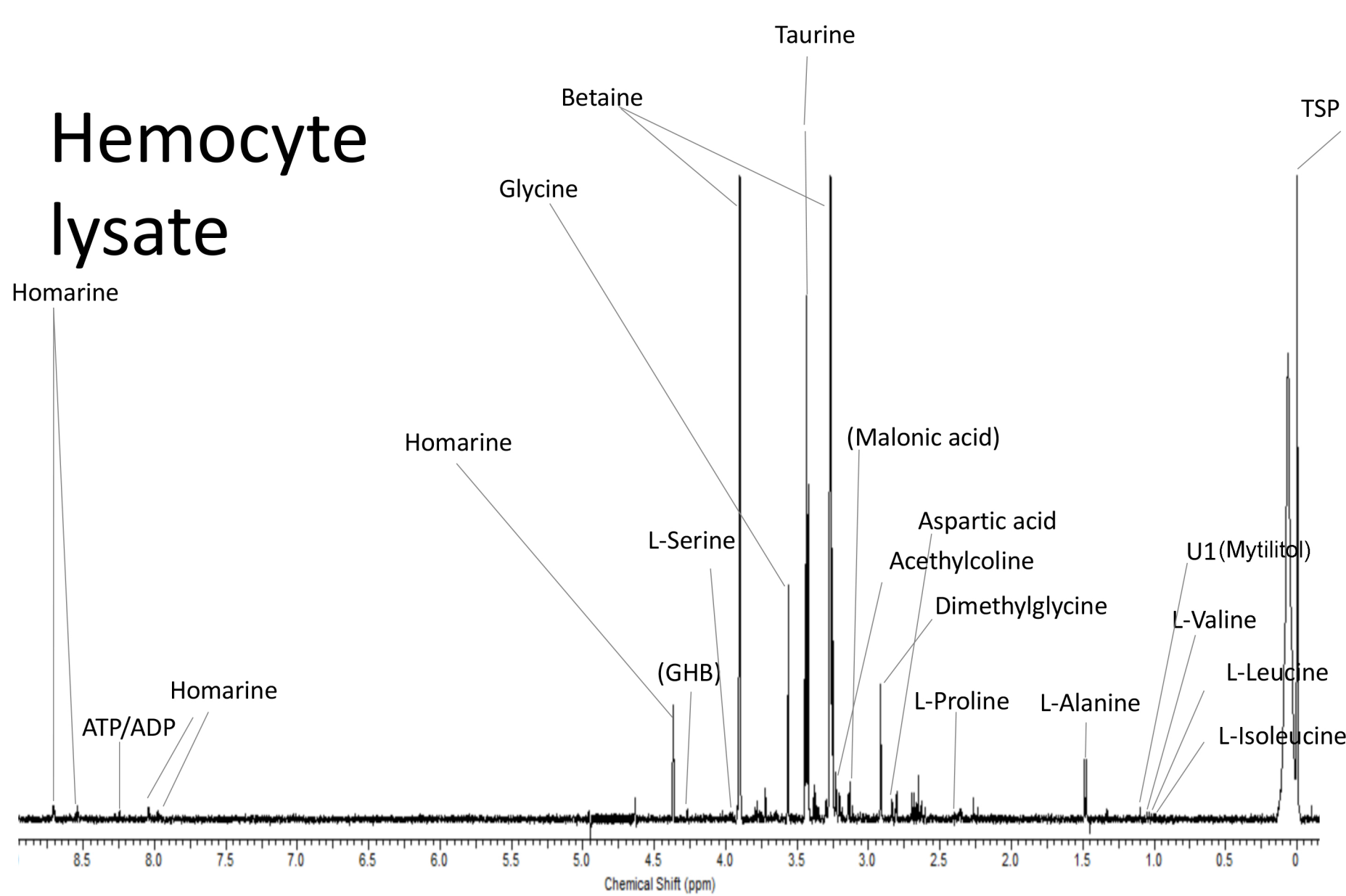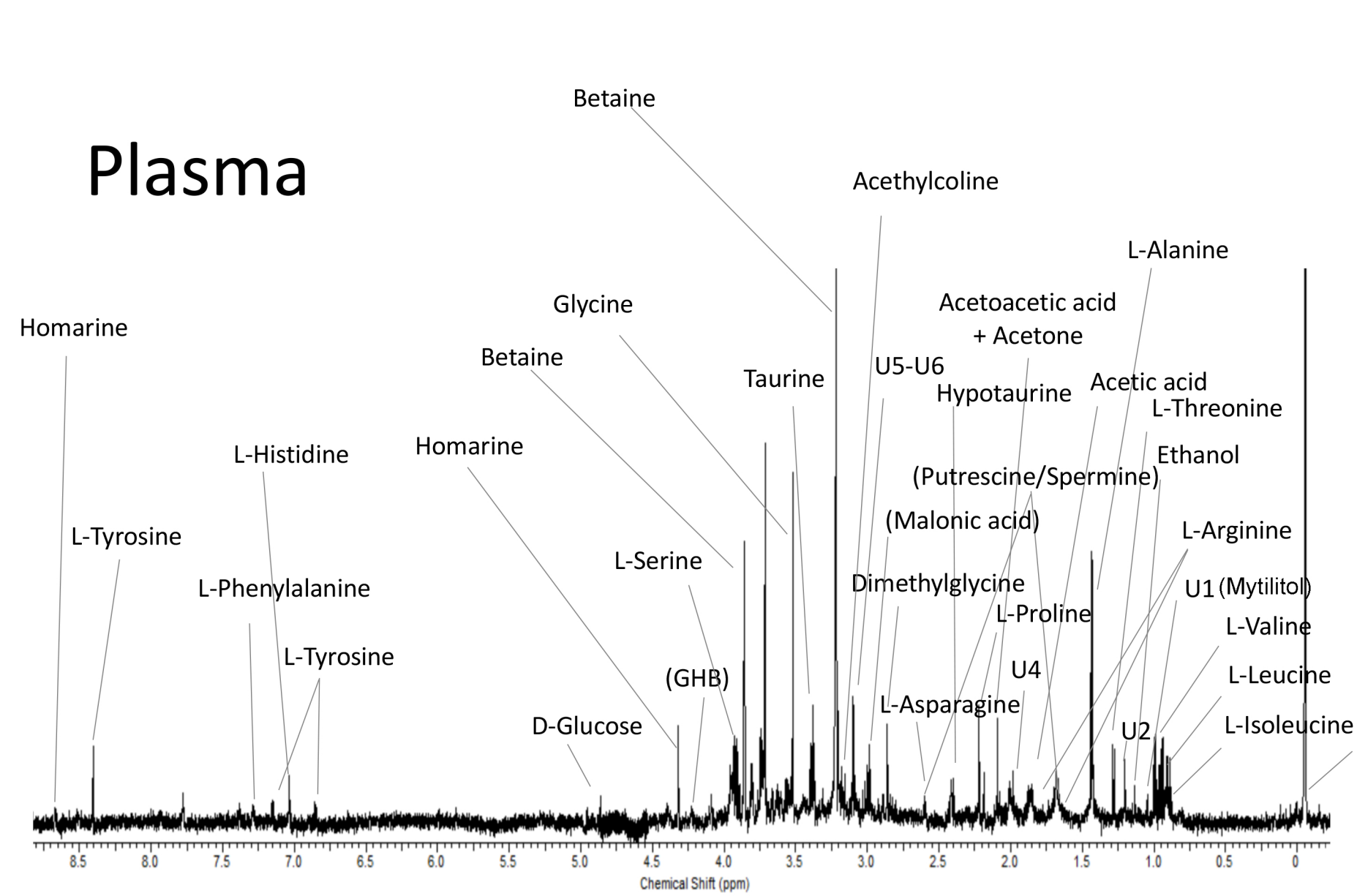

Supplement: Supplementary file 2 [file DataSheet4.pdf]
